# Salmonella infection of an unused fistula

**DOI:** 10.1016/j.jvscit.2025.101861

**Published:** 2025-05-24

**Authors:** Carrie Tackett, Ari Solomon, Jacob Spencer, David Blitzer, John Baber

**Affiliations:** Community Memorial Hospital, Ventura, CA

**Keywords:** Fistula excision, Infected fistula, Salmonella, Unused fistula

## Abstract

When *Salmonella* seeds a vascular structure, it is more frequently associated with mycotic aneurysms of the aorta rather than infected fistulas. A 50-year-old female presented with an infection of her unused arteriovenous fistula. A computed tomography scan of the upper extremity demonstrated dilation of the fistula and signs consistent with a necrotizing soft tissue infection. She underwent an excision of the left upper extremity arteriovenous fistula that was found to have a *Salmonella* infection. Current data suggests that excision of unused fistulas is safe and should be performed if the patient is symptomatic before the development of infection.

Arteriovenous fistulas (AVFs) are the first-line vascular access for patients undergoing chronic hemodialysis, offering better patency and fewer complications than catheters and grafts.^1^^,^^2^ AVFs are critical as reliable, low-risk conduits for repeated blood draws and dialysis sessions, ultimately improving patient survival and quality of life in end-stage renal disease (ESRD).^1^^,^^3^

The majority of AVF-related infections stem from skin flora, including *Staphylococcus aureus* and coagulase-negative *staphylococci*.^4^ Microbes can gain access through needle puncture sites, poor hygiene, or compromised skin barriers, leading to localized infection that can progress to bacteremia.^5^ Although it is common for mycotic aneurysms to be infected with *Staphylococcus aureus* (28%-71%) and *Salmonella* species (15%), it is an uncommon source of fistula infection.^6^ There has been a case of a carotid mycotic aneurysm caused by non-typhoidal *Salmonella*, highlighting the possibility of mycotic aneurysms further away from the gut; however, a *Salmonella* infection of an unused AVF has not been previously reported.^7^

Gram-negative organisms are rarely the causative organisms in AVF infections. *Salmonella* species are generally associated with gastrointestinal illness rather than vascular infections.^8^ When *Salmonella* does infect a vascular structure, it is typically associated with mycotic aneurysms, which are most often seen in the aorta. Infection of an unused, thrombosed AVF by *Salmonella* has not previously been reported in the literature, making it a noteworthy case.^9^ Rare infections such as this one are worthy of documentation, providing clinicians with guidance in early recognition, appropriate antimicrobial therapy, and potential preventive measures for high-risk patients such as unused fistula ligation when appropriate. Written informed consent for publication was obtained from the patient.

## Case report

A 50-year-old female on peritoneal dialysis was found to have a *Salmonella* infection of her unused and thrombosed brachiocephalic AVF. Her past medical history was notable for ESRD on peritoneal dialysis, hypertension, diabetes, and prior stroke. She was previously on hemodialysis, but she had transitioned to peritoneal dialysis several years prior, and her unused AV fistula had subsequently thrombosed a few months prior to presentation, likely secondary to aneurysmal state. The AVF had not been accessed for over 2 years, and she had been taken to the operating room 6 months earlier for excision of her fistula due to pain but went into cardiac arrest on induction of anesthesia, so the operation was aborted.

**Fig 1:**
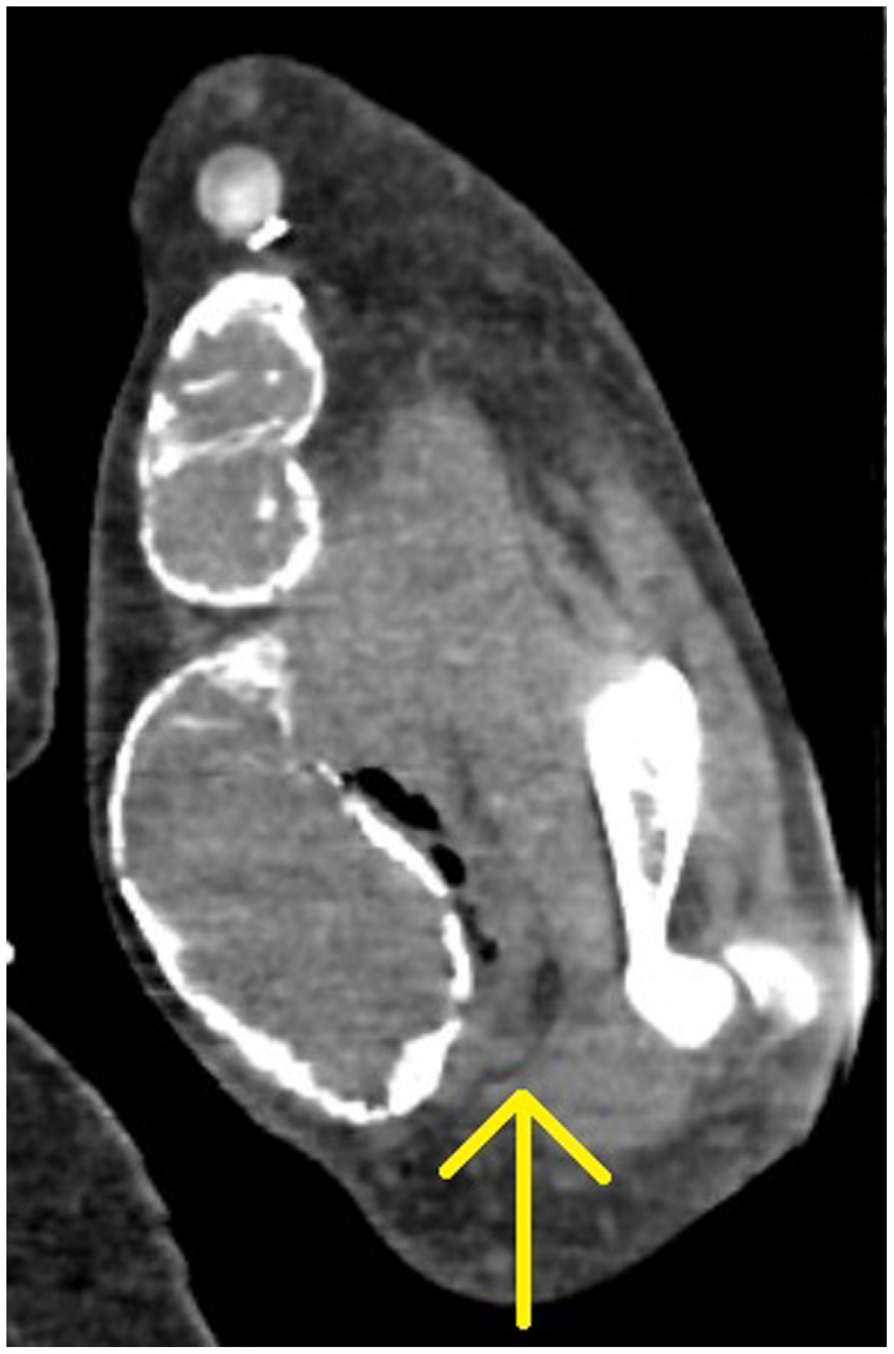
Coronal cut from computed tomography done on admission showing gas and fluid surrounding the patient’s left upper extremity arteriovenous fistula (AVF). Yellow arrow showing fluid/air collection.

**Fig 2:**
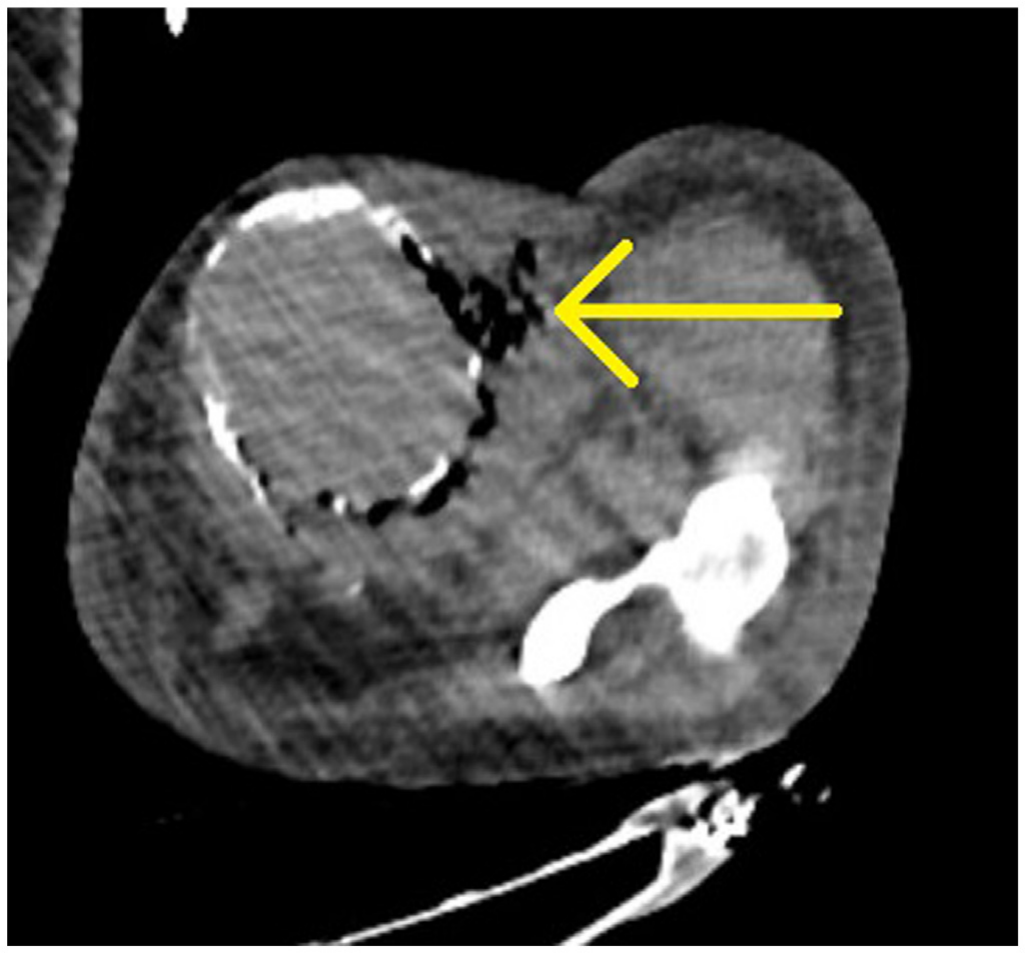
Axial cut from computed tomography done on admission showing gas and fluid surrounding the patient's left upper extremity arteriovenous fistula (AVF). Yellow arrow showing fluid/air collection.

The patient presented to the hospital with 3 days of pain and swelling of her left brachiocephalic AVF as well as fevers and chills. Patient denied any other symptoms such as abdominal pain, nausea, vomiting, or diarrhea. She had no history of immunosuppression, beyond her ESRD. On arrival, she was found to have leukocytosis, tachycardia, and hypotension. A computed tomography scan of the left upper extremity was obtained, demonstrating dilation of the fistula to 4.8 cm and surrounding soft tissue stranding with gas, consistent with a necrotizing soft tissue infection (Figs 1 and 2).

Initial peripheral blood cultures were positive for *Salmonella* species growing from two bottles. Fluid cultures from her peritoneal dialysis catheter had no growth. On exam, she was found to have a thrombosed left upper extremity AVF with erythema and edema of the surrounding tissues. She was taken to the operating room emergently for excision of the AVF and debridement of the surrounding tissues. On the posterior aspect of the fistula, a rupture of the wall was identified with an adjacent soft tissue abscess. The fluid from the fistula and abscess cavity was cultured and grew *Salmonella*. Postoperatively, she was treated with a course of intravenous ceftriaxone based on culture sensitivities. Initially, the patient underwent daily dressing changes until the wound was determined to be stable and free of infection, then a negative-pressure wound dressing was placed to assist in healing. The patient was continued on a 4-week intravenous antibiotic course per infectious disease recommendations for her *Salmonella* infection.

She had several episodes of cardiac compromise during the operation and her postoperative recovery. The remainder of her hospital course included a full cardiac workup, leading to successful percutaneous coronary intervention for coronary artery disease. Due to the multiple sets of negative blood cultures, there was minimal concern about coronary stent placement in the setting of infection. On follow-up, the patient had healed well by 6 weeks after her fistula excision.

## Discussion

Although *Salmonella* is a common cause of mycotic arterial aneurysm, it is unusual for it to occur in an AVF, especially an unused one. Fistulas have a low infection rate of 0.2 to 0.4 per 1000 days, whereas prosthetic grafts have a 10-time higher infection rate. Risk factors for fistula infections include poor hygiene, diabetes, skin wounds, and buttonhole cannulation.^5^ In this case, the patient’s fistula had not been used in over 2 years as she had been undergoing peritoneal dialysis. The patient was diabetic, but she did not have a wound in the area before the development of her symptoms.

Although anatomically different from an AVF, it is notable that mycotic arterial aneurysms have been commonly associated with *Salmonella,* with it being identified as the causative organism in 15% of cases.^6^ Different pathophysiological theories behind these aneurysms include infection, bacteremia, local injury and spread, and septic emboli.^6^ In this case, the patient had *Salmonella* bacteremia, which we believe was the likely source of the fistula infection via hematogenous spread.

There is only one previously documented case of an AVF infected with *Salmonella*.^9^ In contrast with our case, this patient’s fistula was being accessed regularly for dialysis use, and the fistula infection was attributed to an outbreak of *Salmonella* in the hemodialysis unit the patient was visiting multiple times weekly.^9^

Current data suggests that excision of unused fistulas is safe and should be performed if the patient is symptomatic,^10^ but there is a debate over whether it is prudent to excise or ligate noninfected fistulae when they are not in use and asymptomatic. In the case of this patient, excision was attempted 6 months prior due to reported discomfort at the fistula site, but the patient had suffered from cardiac collapse on induction of anesthesia, so surgery was aborted.

## Conclusions

This is only the second case of *Salmonella*-related AVF infection documented in the literature. It is very unusual to have an infected, unused AVF, and even more unusual to find *Salmonella* as the causative organism. In this case, the common risk factors, such as skin breakdown or cannulation of the fistula, were not present, showing that even unused fistulae are at risk for complications.

## Funding

None.

## Disclosures

None.
